# Comparative Genome Analysis of Hungarian and Global Strains of *Salmonella* Infantis

**DOI:** 10.3389/fmicb.2020.00539

**Published:** 2020-04-03

**Authors:** Tibor Nagy, Ama Szmolka, Tímea Wilk, János Kiss, Mónika Szabó, Judit Pászti, Béla Nagy, Ferenc Olasz

**Affiliations:** ^1^National Agricultural Research and Innovation Centre, Agricultural Biotechnology Institute, Gödöllő, Hungary; ^2^Centre for Agricultural Research, Institute for Veterinary Medical Research, Budapest, Hungary; ^3^National Center for Epidemiology, Budapest, Hungary

**Keywords:** *Salmonella* Infantis, whole-, core-, and accessory genomes, antibiotic resistance genotypes, genomic-, and pathogenicity islands, outlier *S*. Infantis isolates

## Abstract

**Introduction:**

The emergence and spread of new strains of zoonotic bacteria, such as multidrug resistant (MDR) *Salmonella* Infantis, represent a growing health risk for humans in and outside Europe due to foodborne infections of poultry meat origin.

**Objectives:**

In order to understand genome relations of *S*. Infantis strains from Hungary and from different geographic regions, we performed a comprehensive genome analysis of nine Hungarian and 67 globally selected strains of *S*. Infantis and 26 *Salmonella* strains representing 13 non-Infantis serovars.

**Results:**

Analyses of whole-, and accessory genomes, showed that almost all *S*. Infantis strains were separated from the non-Infantis serovars. *S*. Infantis strains from Hungary formed subclusters based on their time of isolation. In whole genome sequence analysis, the Swiss strains of *S*. Infantis were closely related to each other and clustered together with subclusters of strains from Hungary, Japan, Italy, United States, and Israel. The accessory genome analysis revealed that the Swiss strains were distinct from most of the strains investigated, including the Hungarian ones. Analysis of the cloud genes offered the most detailed insight into the genetic distance and relationship of *S*. Infantis strains confirming that the Swiss and Hungarian strains belonged to different lineages. As expected, core genome analysis provided the least discriminatory power for analysis of *S*. Infantis. Genomic sequences of nine strains from Brazil, Israel, Mexico, Nigeria, and Senegal (deposited as *S*. Infantis) proved to be outliers from the *S*. Infantis clade. They were predicted to be *Salmonella* Rissen, *Salmonella* Ouakarm, *Salmonella* Kentucky, *Salmonella* Thompson, and *Salmonella* enterica subsp. *diarizonae*.

**Conclusion:**

Accessory genome of *S*. Infantis showed the highest diversity suggesting a faster evolution than that of the whole genomes contributing to the emergence of multiple genetic variants of *S*. Infantis worldwide. Accordingly, in spite of the comprehensive analysis of several genomic characteristics, no epidemiologic links between these *S*. Infantis strains from different countries could be established. It is also concluded that several strains originally designated as *S*. Infantis need in databanks reclassification.

## Introduction

For several decades, non-typhoidal salmonellosis has become the first or second most frequent foodborne zoonosis worldwide with *Salmonella* Enteritidis as the leading serovar, followed by serovars Typhimurium (especially of monophasic *Salmonella* Typhimurium) and *Salmonella* Infantis, which have increased during the last decade in humans. The primary sources of *S*. Enteritidis infections are layers and layed eggs while those of *S.* Infantis infections are broiler chicken (European Food Safety Authority [EFSA] and European Centre for Disease Prevention [ECDC], 2017a, b, 2019). In spite of the implementation of *Salmonella* monitoring and control programs in the EU and in contrast to the earlier declining trend of human salmonellosis (O’Brien, 2013), there have been some unfavorable changes on this trend since 2016 in Europe. Especially worrying is that the prevalence of *S*. Infantis infections has almost doubled among humans in the last few years. About 90% of such human cases were of broiler origin. Furthermore, recent isolates of *S*. Infantis show increasing prevalence and diversity of antibiotic resistance (European Food Safety Authority [EFSA] and European Centre for Disease Prevention [ECDC], 2017a, b, 2019). As a consequence, we are witnessing an increased public awareness and increasing health concerns due to *S.* Infantis infection of broilers in Europe.

*Salmonella* Infantis appears to be an emerging serovar worldwide as well. It has been reported as the most frequently isolated serovar from fresh poultry meat and broiler flocks as well as from human in Japan (Asai et al., 2006; Shahada et al., 2006; Murakami et al., 2007), Belgium, France (Cloeckaert et al., 2007), and Hungary (Nógrády et al., 2007, 2008). *S.* Infantis has also emerged in Israel and became the leading serovar in humans and broilers (Gal-Mor et al., 2010). Similar trend has been reported in Italy (Dionisi et al., 2011). The first reports about the emergence and clonal spread of multidrug resistant (MDR) *S*. Infantis in human populations and in broilers in several European countries came from Nógrády et al. (2007, 2008). The emerging “clone B” strains, as determined by pulsed field gel electrophoresis (PFGE), were predominantly resistant for nalidixic acid, streptomycin, sulfonamides, and tetracycline (NSSuT) and carried a conjugative ∼270 kb MDR plasmid pSI54/04 containing a class 1 integron with *aad*A1 gene cassette and the *tet*(A) gene (Nógrády et al., 2007, 2008, 2012; Olasz et al., 2015; Szmolka et al., 2018). Sequence analysis and PCR-typing of plasmid pSI54/04 revealed a high sequence similarity to the megaplasmid pESI of human *S*. Infantis from Israel (Aviv et al., 2014). However, both *in vivo* and *in vitro* evidence for the pathogenic significance of this plasmid in chicks is missing (Szmolka et al., 2018). In order to obtain underlying genomic data for subsequent analysis of the relations of *S*. Infantis clones in Hungary, we have performed whole genome sequencing (WGS) projects (Olasz et al., 2015; Wilk et al., 2016, 2017).

Bioinformatic analyses of sequence data from our projects and those available in different databases provided a promising approach to obtain new information and in-depth insights into *Salmonella* genomics. In case of *S*. Infantis, the amount of relevant data available in public databases and genomic studies increased significantly in the past few years (Yokoyama et al., 2014; Franco et al., 2015; Hindermann et al., 2017; Acar et al., 2019). However, the type, genome status, and quality of these datasets are usually diverse depending on the intention of the depositors. Sequence data deposited in the NCBI GenBank include reads or contigs, scaffolds, or even whole genomes and contain relevant information on the completeness of the sequence, which was required to select suitable data set for our analyses.

So far genomic studies on *S*. Infantis have primarily dealt with sequences from national collections and studied genomic relations between strains of the respective countries, i.e., Japan, Italy, and Switzerland (Yokoyama et al., 2014; Franco et al., 2015; Hindermann et al., 2017; Acar et al., 2019). In order to understand genomic relations of *S*. Infantis strains from different geographic origin, here we performed a comprehensive genome analysis of nine Hungarian and further 67 globally selected *S.* Infantis and 26 other *Salmonella* strains representing 13 non-Infantis serovars. Comparisons of the whole-, core-, accessory genomes allowed us to obtain a more comprehensive view about possible genomic relations of *S*. Infantis from different countries and geographic regions.

## Materials and Methods

### Selection and Origin of *Salmonella* Genome Sequences

For the analyses, whole genome sequence of 76 *S.* Infantis strains was selected. The isolates were selected on the basis of already published papers. The concept of the choice of the isolates was as follows: (i) Isolates from a single country were preferred if published in a single publication (see Supplementary Table S1). (ii) Each country was represented by a maximum 10 strains of poultry (food) or human (clinical) origin if possible. (iii) Further criterion for the selection was the time of publications (between 2014 and 2017 with the exception of Senegal SARB27, which was published earlier; Boyd et al., 1993), representing recent isolates. (iv) Beyond that, the choice of the sequences of the isolates was random.

Among the 76*S.* Infantis strains, nine strains derived from Hungary and the other 67 strains were selected from diverse European and non-European countries. Hungarian strains covered two periods of isolation, and were designated here as pre-emerging (1980–1994) and emerging (2000–2016) strains. The latter group representing the emergence of MDR *S*. Infantis in Hungary (Nógrády et al., 2007; Olasz et al., 2015; Wilk et al., 2016, 2017). The annotated genome of the earliest sequenced *S*. Infantis strain UK-1973 1326/28 was included as reference.

In addition to *S*. Infantis, genome sequences of 26 *Salmonella* strains representing 13 most common *Salmonella* serovars in animals and humans based on the paper of Zou et al. (2013) were also included into the analyzes. Each of these serovars was represented by two strains, except Paratyphi, where three strains were included for the biovars A, B, and C (Supplementary Table S1). The sequence of *Salmonella bongori* strain NCTC12419 was used as a non-*enterica* control (Fookes et al., 2011). In this way, a total of 102 *Salmonella* genomes were downloaded from the NCBI database (Agarwala et al., 2016). Raw sequences deposited in SRA database (Leinonen et al., 2011) were assembled into contigs using the SPAdes v.3.11.1 program (Bankevich et al., 2012) with default settings. Contigs were annotated using Prokka v1.14.1 with default settings (Seemann, 2014).

### Bioinformatics and Phylogenetic Analysis

Core- and accessory genome analyses were performed with Roary (Page et al., 2015). The input files were produced by Prokka and the core gene alignment method was set to MAFFT. IslandViewer4 (Bertelli et al., 2017) was used to identify genomic islands (GIs) in the reference strain UK-1973 1326/28. *Salmonella* pathogenicity islands (SPIs) were also defined according to this reference strain.

Blast + (Camacho et al., 2009) was used for identification of the GI, SPI genes, as well as of core-, accessory-, and flagellin genes. The identification criteria for GIs were the following: the region should be covered 90% and the similarity should be larger than 90%. ProgressiveMauve with default settings was used to perform multiple alignments with default settings (Darling et al., 2010). Dendroscope 3.6.3 (Huson et al., 2007) was used to draw the final trees from Mauve’s guided tree with rectangular phylogram option. The whole genome analysis—including the custom Python scripts is available at this link: https://github.com/TravisCG/articles/tree/master/infantis2020/. The serovar-marker flagellin gene *flj*B of *S*. Infantis was identified according to Kardos et al. (2007). The flagellin genes *flj*A, *flj*B, *fli*A, *fli*B, *fli*C, *fli*D, *fli*S, and *hin* were identified based on sequences of the reference strain (Liu et al., 2017). For *in silico* prediction of the serovar and clonal identity of the strains, the web-based softwares SeqSero and MLST were used (Larsen et al., 2012; Zhang et al., 2015). The acquired antibiotic resistance genes of *S*. Infantis were identified by using the ResFinder v3.2 (Zankari et al., 2012). Selected thresholds for the prediction of resistance genes were: identity 90%; minimum length 80%. The assembled contigs were used as input sequence for the *in silico* prediction programs.

## Results

### Analysis of Whole Genome Sequences

**FIGURE 1:**
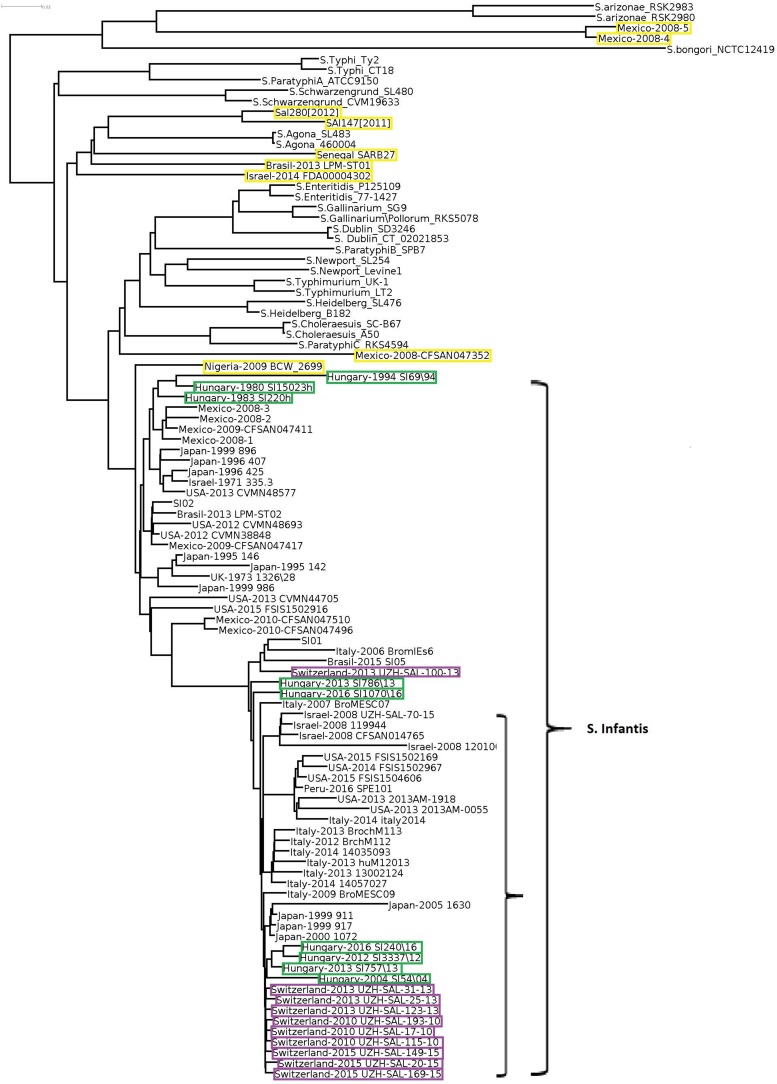
Whole genome tree of all selected *Salmonella* strains including nine Hungarian and 67 global *S*. Infantis strains and 26 strains of 13 non-Infantis *Salmonella* serovars. Green boxes show the pre-emerging and the recent Hungarian isolates; purple boxes indicate Swiss strains, while yellow boxes indicate the *Salmonella* strains originally deposited as *S.* Infantis but outlying from the Infantis cluster. The main *S*. Infantis subclade is indicated. The bar represents average similarity between the genomes corresponding to genetic distance. These signs were consequently applied to all figures. *S. bongori* was used as outgroup control.

In order to obtain detailed information on relationships of the 102 selected *Salmonella* strains (Supplementary Table S1), whole genome sequences were compared, including the two *S. enterica* subsp. *arizonae* and the *S. bongori* strains as internal subspecies- and species controls. The genomic reconstruction showed that the nine Hungarian *S*. Infantis strains grouped in three subclusters according to their time of isolation (Figure 1). One cluster contained two subclusters representing six recent (emerging) strains (2004–2016), while the other included three pre-emerging strains isolated between 1980 and 1994. This observation was in good agreement with our results obtained by PFGE (Supplementary Figure S1). In general, some geographic clustering of *S*. Infantis strains was recognized. The genomes of nine Swiss strains from 2010 to 2013 proved to be closely related to each other and formed a separate subcluster and clustered together with subclusters of strains from Hungary, Japan, Italy, United States, and Israel.

*Salmonella* Infantis strains formed a large well-defined clade clearly separated from the non-Infantis serovars in the whole genome analysis, with the exception of nine strains. Out of these, the strains Mexico-2008-4 and -5 formed a divergent outlier cluster, which was a sister group of *S*. *enterica* subsp. *arizonae*. Six other outlier *S*. Infantis, Sal280[2012], Sal147[2011], Senegal SARB27, Brasil-2013 LMP-ST01, and Israel-2014 FDA00004302 grouped together with *Salmonella* Agona, while Mexico-2008-CFSAN047352 was most closely related to *Salmonella* ParatyphiC_RKS4594. The strain Nigeria-2009 BCW_2699 showed moderate similarity with the *S*. Infantis clade. The non-Infantis strains clustered separately in pairs according to their serovars (Figure 1).

### Analysis of the Core- and Accessory Genomes

Genomes were analyzed according to the genomic units as determined by Inglin et al. (2018). Core genes, representing genes present in all selected genomes, are generally responsible for fundamental functions such as metabolism, basic regulation of cell cycle, maintenance of the genome, or cell envelope. Softcore genes are present in 95–99% of the strains. The accessory genome includes genes that are present occasionally in several strains. These are often responsible for strain specific traits and can be divided into the classes of shell genes that are present in a remarkable proportion of the strains (15–95%) and cloud genes that are unique or specific to a smaller fraction of strains (<15%) (Table 1).

**TABLE 1:** Distribution of core and accessory genes according to the tested set of Salmonella strains.

Group	Core genes (99–100%)	%	Soft core genes (95–99%)	%	Shell genes (15–95%)	%	Cloud genes (<15%)	%	Whole genome (100%)	%
All *Salmonella* (*n* = 102)	2030	10,8	969	5,1	1924	10,2	13924	73,9	18847	100
All *S.* Infantis (*n* = 76)	2316	18,0	1268	9,8	1132	8,8	8175	63,4	12891	100
*S*. Infantis Hungary (*n* = 9)	4123	78,8	0	0	711	13,6	395	7,6	5229	100
Hungary + Swiss (*n* = 19)	4053	75,0	0	0	670	12,4	684	12,7	5407	100
Hungary + United States (*n* = 19)	3968	65,2	0	0	898	14,8	1220	20,0	6086	100
Hungary + Japan (*n* = 19)	3863	65,5	0	0	1014	17,2	1020	17,3	5897	100
Hungary + Italy (*n* = 19)	4102	72,3	0	0	648	11,4	920	16,2	5670	100
Swiss + Italy (*n* = 20)	4139	77,6	82	1,538	438	8,2	672	12,6	5331	100
Swiss + Japan (*n* = 20)	3869	69,1	223	3,984	715	12,8	790	14,1	5597	100
Swiss + United States (*n* = 20)	3975	68,8	127	2,198	681	11,8	996	17,2	5779	100
Italy + Japan (*n* = 20)	3887	66,7	228	3,911	727	12,5	988	16,9	5830	100
Italy + United States (*n* = 20)	4004	67,2	115	1,93	668	11,2	1171	19,7	5958	100
Japan + United States (*n* = 20)	3814	61,7	238	3,849	912	14,8	1219	19,7	6183	100

The pan-genome of the 102 *Salmonella* strains contained 18,847 genes, while 2030 genes were identified as core genes (Table 1 and Supplementary Table S2). In the 76 *S*. Infantis strains investigated, the number of core genes was a bit higher (2316) and the number of cloud genes was lower (8175). In Table 1, the number and distribution of the core, soft core, shell, and cloud genes in *S*. Infantis strains and their percentual overlap from different countries (Hungary, Switzerland, United States, Japan, and Italy) were also compared. The number of core genes in the studied countries is very similar (3814–4139) and no major geographical differences were observed. The number of soft core genes is low (0–238), while the number of shell and cloud genes is more variable (shell genes: 438–1014; cloud genes: 684–1219).

**FIGURE 2:**
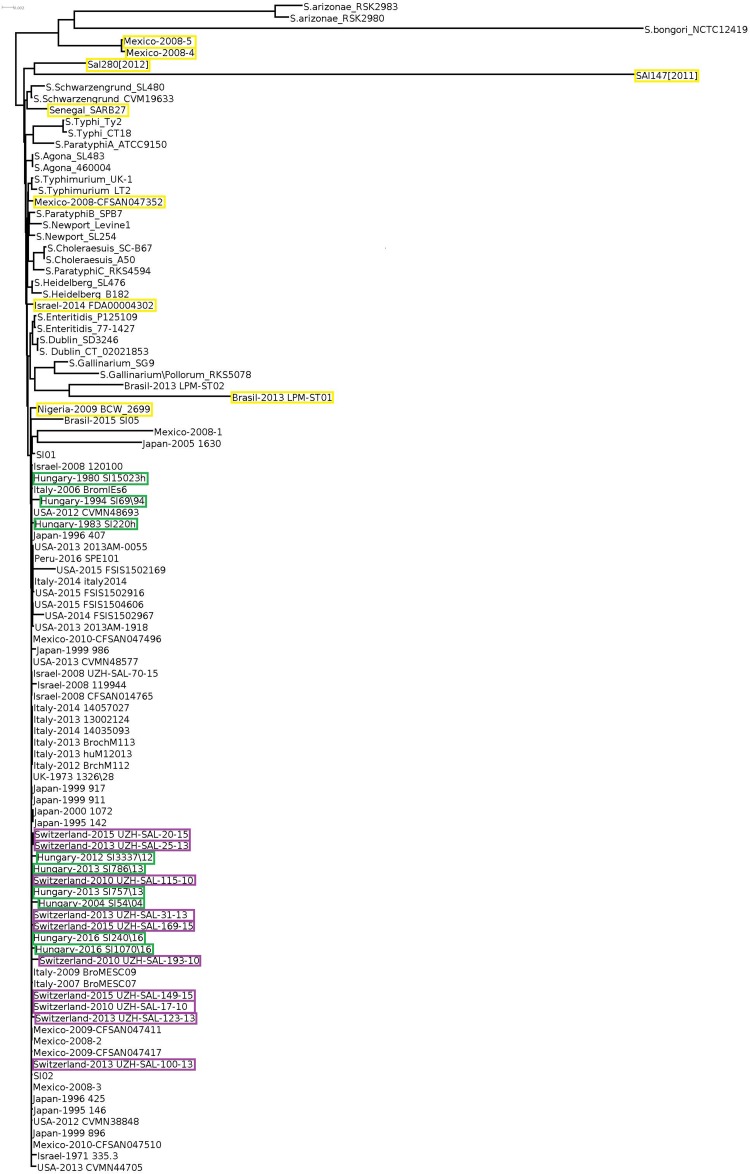
Core genome tree of all *Salmonella* strains investigated. Yellow boxes indicate the *Salmonella* strains outlying from the *S*. Infantis cluster. Only the strains from Hungary, Switzerland, and the outlier *S*. Infantis isolates are highlighted by color boxes.

Core genome-based phylogenetic reconstruction of these 102 *Salmonella* strains showed that a large set of *S*. Infantis strains clustered separately from the non-Infantis strains without indication of *S*. Infantis subclusters (Figure 2). The nine outliers of the Infantis clade appeared in different branches of the core-genome tree together with the non-Infantis strains. The separate core genome analysis of *S*. Infantis strains did not show significant diversity of these strains either, except for the outlier strains as pointed out above (Supplementary Figure S2).

**FIGURE 3:**
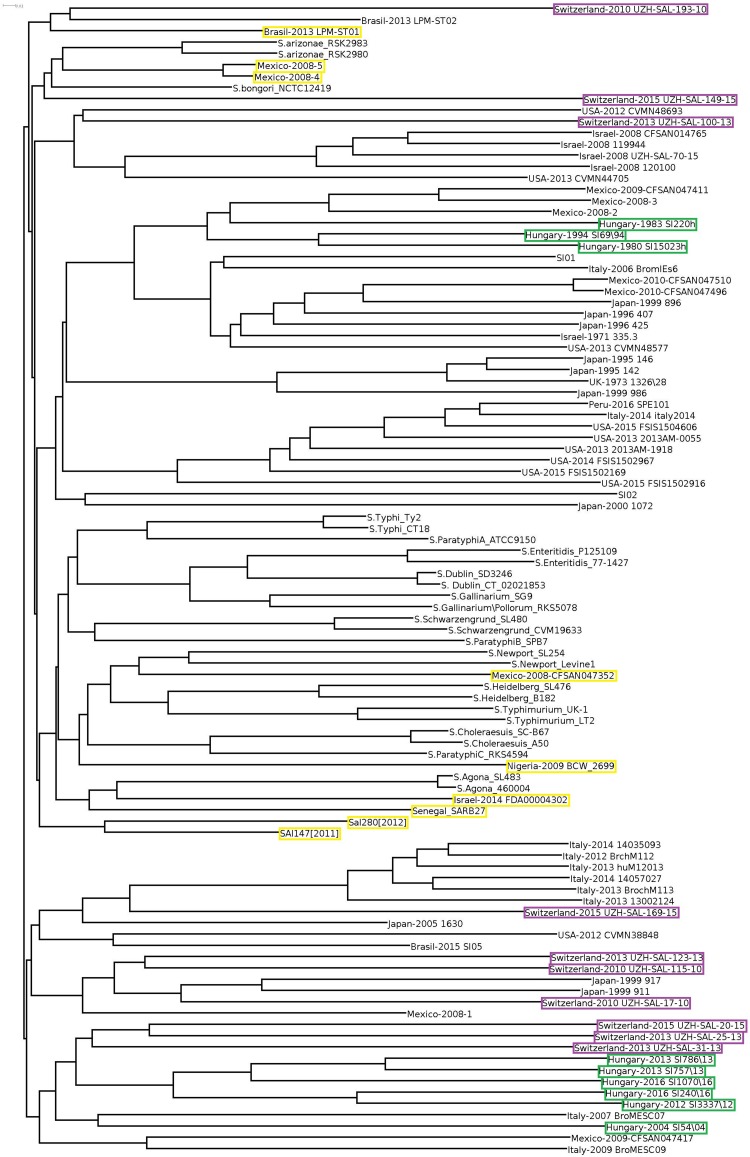
Cloud-gene-based tree of all *Salmonella* serovars. The outlier *S*. Infantis isolates are indicated by yellow boxes, while strains from Hungary and Switzerland are highlighted by green and purple boxes, respectively.

Cloud gene tree of the 102 *Salmonella* genomes reflected the separation of *S*. Infantis and non-Infantis strains in a way that *S*. Infantis strains were divided into two large clades by a third clade of non-Infantis serovars (including four outlier strains) in the central position (Figure 3). The Hungarian and Swiss strains formed separate well identifiable clusters in both clades of S. Infantis. Besides, the Hungarian strains consistently exhibited the time-related separation (pre-emerging and recent isolates) as it was observed in WGS analysis (Figure 1). Furthermore, one of the Swiss strains (Switzerland 2010 UZH-SAL-193-10) branched further away from the main clade together with one Brazilian strain of *S*. Infantis. Besides, the outliers from Mexico (Mexico-2008-4 and Mexico-2008-5) were positioned also near to this cluster together with *S*. *bongori* and *S*. *arizonae* (Figure 3).

**FIGURE 4:**
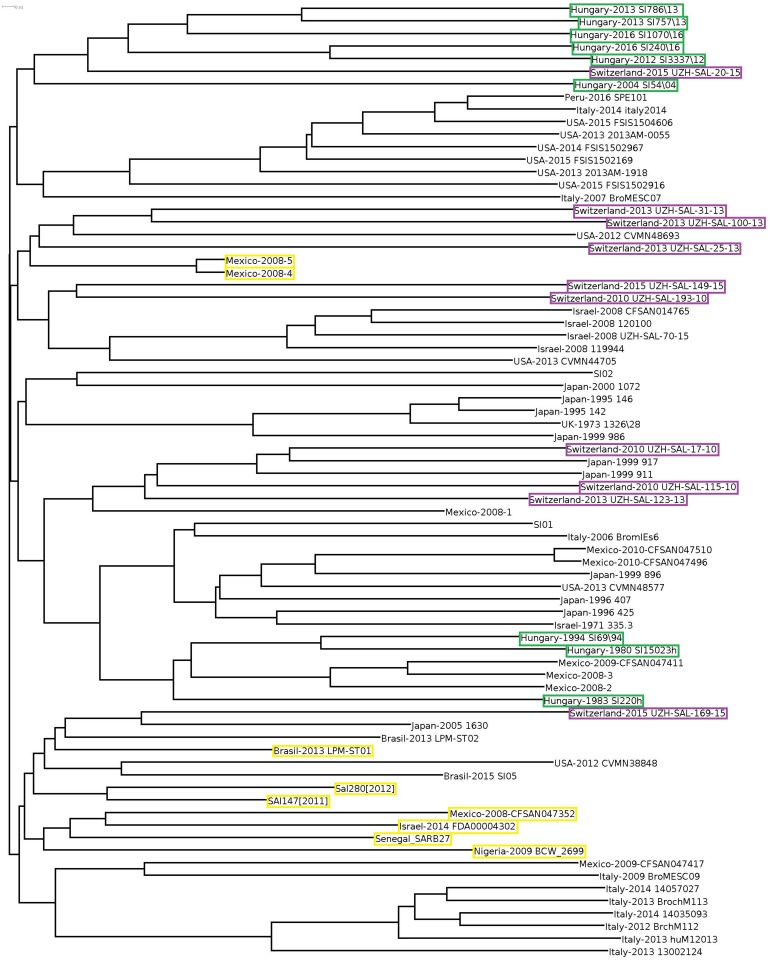
Cloud-gene-based tree of *S.* Infantis strains. The marks and symbols are as in Figure 1. Only the strains from Hungary, Switzerland, and the outlier *S*. Infantis isolates are highlighted by color boxes.

When cloud genes of the *S*. Infantis strains were analyzed, their distribution appeared much more diverse (Figure 4). The Hungarian strains consistently exhibited the time-related separation observed previously. One large cluster of six Hungarian emerging strains also included one Swiss strain (Switzerland-215-UZH-SAL-2015) representing the only Swiss strain with genetic relatedness to Hungarian strains. The Swiss isolates did not form a distinct cluster but they were separated into four subclusters grouped together with some strains from Italy, Israel, and Japan, showing no close genomic relation to either the recent or the pre-emergent Hungarian strains in this cloud gene-based analysis (Figure 4). The outlier Infantis strains Senegal SARB27, Mexico-2008-4 and -5, Brasil-2013 LPM-ST01, Sal280[2012] and SAl147[2011], Israel-2014 FDA00004302, Mexico-2008-CFSAN047352, and Nigeria-2009 BCW_2699 were located separately. The congruent results of the whole-, core-, and cloud-genome-based analyses imply that these outlier strains do not belong to the serovar Infantis (Figures 3, 4).

### Antibiotic Resistance Genotype of *S*. Infantis Strains

Out of the 76 *S*. Infantis strains tested, 45 were predicted as multiresistant on the basis of the co-existence of at least three resistance genes (Supplementary Table S3). According to this, the association between genes *tet*(A), *sul1*, and *aadA1* was found in 64.4% of the MDR strains. In some strains from the United States and Italy, a high abundance of antibiotic resistance genes was detected including ESBL genes *bla*_CTX–M–__65_ or *bla*_CTX–M–__1_. Multiresistance genotypes were more characteristic to the recent strains, isolated between 2000 and 2016, while most of the old isolates (years 1971–1999) did not carry acquired antibiotic resistance genes. Examining the geographical distribution of resistance genes, we found that no strain isolated from Mexico exhibited antibiotic resistance, unlike strains isolated in other countries.

### Analysis of Flagellin Genes of *Salmonella* Serovar Infantis

The presence of *flj*A, *flj*B, *fli*A, *fli*B, *fli*C, *fli*D, *fli*S, and *hin* flagellin- or flagella related genes was tested in all 102 selected *Salmonella* strains. The *fli*A,B,D,S genes were detected in almost all serovars represented here (Supplementary Table S4). Most strains of *S*. Infantis were characterized by the co-occurrence of all the above flagellar genes. Exceptions were the strains Hungary-2013 SI757/13, United States-2014 FSIS1502967, 7 strains from Japan, Mexico-2008-1, and Brasil-2013 LPM-ST02 which showed the absence of one of the genes. Three of the outlier strains, Israel-2014 FDA00004302, Senegal SARB27, and Nigeria-2009_BCW_2699 harbored all the eight flagellar genes; however, they were consistently separated from the Infantis clusters in all phylogenetic analyses (Figures 1–3). The other six outlier *Salmonella* isolates were deficient in several flagellar genes that further strengthened the need for validation of the serovar on the basis of genomic sequences as well.

Results on the *in silico* prediction of the antigenic profiles (O:H1:H2) and of the MLST profile of the above nine outlier *Salmonella* strains are presented in Table 2. According to this, the Senegal SARB27 (ST79) was the only strain that was predicted as *S*. Infantis, but it was assigned to ST79 instead of ST32 characteristic for *S*. Infantis. Two strains, Sal147[2011] and Sal280[2012], were identified as *S*. Rissen (7:f,g:-) both belonging to ST469. Further tree strains deposited as *S*. Infantis were predicted as Kentucky, Thompson, and Ouakarm assigned to ST198, ST26, and ST1610, respectively. The Mexican isolate Mexico-2008-4 was predicted to be *S*. *enterica* subsp. *diarizonae* with the antigenic profile of 60:r:e,n,x,z_15_ and ST63. For two outlier strains (Mexico-2008-5 and Nigeria-2009 BCW_2699), there was not possible to detect the serovar on the basis of genomic sequences (Table 2). All other *S*. Infantis strains proved to be ST32 including those that were regarded as Infantis-like in Table 2. The only exception was the serovar Gege (ST36).

**TABLE 2:** Serovars and sequence types (STs) of the outlier *Salmonella* Infantis isolates on the basis of *in silico* prediction.

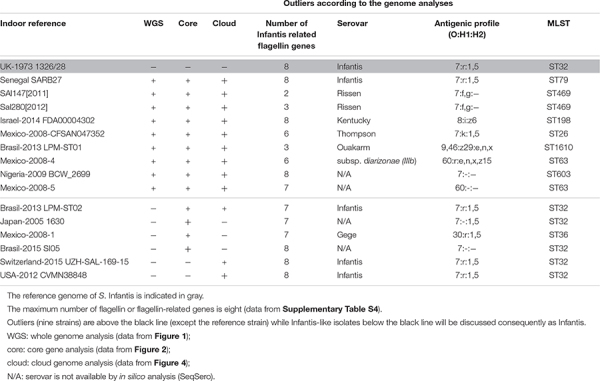

### Identification and Analysis of Genomic and Pathogenicity Islands

In order to reveal the prevalence and diversity of GIs of *Salmonella*, the first annotated complete *S*. Infantis genome UK-1973 1326/28 (Olasz et al., 2015) was used as a reference to determine their number, location, length and coding capacity (Supplementary Table S5—under tab “1326_28 island summary” and “1326_28 island annotation,” respectively). In the genome of the reference strain, 52 segments with coding capacity for 633 annotated genes were detected as parts of putative GIs whose length ranged between 4082 and 47,931 bp (Supplementary Table S5). The genes *avr*A and *sop*E2 characteristic for SPI-1 were found on the predicted islands gi.20 and gi.23, respectively, and the SPI2-specific *spi*C and *ssa*Q gene was located on island gi.19 and gi.10, respectively, indicating that the prediction method applied was able to identify the well-known SPIs, i.e., SPI-1 and SPI-2.

Using the sequences of the 52 predicted islands (GIs and SPIs), their prevalence was tested in all *S*. Infantis strains (Supplementary Table S6). The presence of a particular island varied between 5 and 76 occurrences with the average of 70.9 (93.2%) among the 76 genomes. It also should be mentioned that the outlier strains Brasil-2013_LPM-ST01, Senegal_SARB27, SAl147[2011], Sal280[2012] Mexico-2008-4, and -5, harbored less than 83% of the 52 predicted islands (Supplementary Table S6), indicating their divergent evolutionary lineages.

## Discussion

Over the past decades, *S.* Infantis has become more prevalent among broiler flocks, representing an increasing health hazard for humans in Europe, Israel, Japan, and United States. Accordingly, *S.* Infantis strains of humans and poultry have been studied worldwide (Shahada et al., 2006, 2010; Cloeckaert et al., 2007; Nógrády et al., 2007, 2008, 2012; Gal-Mor et al., 2010; Dionisi et al., 2011; Tate et al., 2017) and a growing portion of strains proved to be MDR. So far, only few genomic analyses have been reported on different *Salmonella* serovars (Zou et al., 2013) and *S.* Infantis strains (Yokoyama et al., 2014), with special regard to MDR *S*. Infantis of the respective countries (Franco et al., 2015; Hindermann et al., 2017; Szmolka et al., 2018; Acar et al., 2019). Here we provide a comprehensive analysis about the possible relations between *S.* Infantis strains from different countries and between *S.* Infantis and other non-Infantis serovars. For this, we applied whole-, core-, and accessory genome analyses.

Whole genome comparisons showed that a tendency for geographic origin was reflected in clustering of *S*. Infantis isolates from countries represented by 9–10 isolates from Switzerland, Hungary, Japan, Italy, and United States (Figure 1 and Table 2). This may indicate the differences in contamination and re-contamination of production units from their environments together with the differing local practices of the antibiotic usage which could influence the selection and persistence of various clones and subclones of *S.* Infantis.

Our data on antimicrobial resistance confirm the recent global emergence of resistance of S. Infantis. From the presented data cannot be concluded that the spread of a single clone could be responsible for the wide spread of antibiotic resistance, but it is more likely to be due to multiple simultaneously appearing clones with similar resistance patterns (Gal-Mor et al., 2010; Franco et al., 2015; Tate et al., 2017; Acar et al., 2019; Bogomazova et al., 2019).

In this study, the nine Hungarian isolates showed a time-related separation as well: the pre-emerging and recent isolates formed two distinct clusters. Furthermore, nine out of 10 genomes of recent Swiss isolates also formed a single cluster in WGS analyses (Figure 1). Our results (Figures 1, 2, 4) demonstrated that the core genome analysis as performed by Hindermann et al. (2017) is not discriminative enough to determine whether or not the *S.* Infantis strains from Switzerland are really clustered together with, and could be epidemiologically related to strains from Hungary, Japan, Italy, United States, and Israel. Our results also indicated that the analysis of core genomes provides less detailed information about the different genetic lineages as discussed by Pornsukarom et al. (2018). In this respect, the phylogenetic reconstructions based on cloud genes (Figures 3, 4) remarkably differ also from that based on the whole genomes (Figure 1). According to our cloud genome analysis, the recent Hungarian isolates represent a cluster being separated not only from the Swiss isolates but also from most of the European and global isolates (Figures 3, 4). Therefore, the core genome-based suggestion of Hindermann et al. (2017) that the Hungarian MDR clone of *S.* Infantis would be documented in Swiss food and human strains and it would have “spread within and outside Europe” seems to be unsubstantiated. Our present data and those from the literature (Gal-Mor et al., 2010; Franco et al., 2015; Tate et al., 2017; Acar et al., 2019) indicate that some *S*. Infantis clones may emerge and persist in different geographic areas almost simultaneously, suggesting that vertical dissemination of certain clones within the poultry sector should also be considered.

All of our genome analyses suggested that nine of the 76 strains (Sal147[2011], Sal280[2012], Israel-2014 FDA00004302, Mexico-2008-CFSAN047352, Brasil-2013 LPM-ST01, Mexico-2008-4, Mexico-2008-5, Nigeria-2009 BCW_2699, and Senegal SARB27) previously identified as *S*. Infantis are distant from the main Infantis clade and could represent other serovars, except the strain Senegal SARB27. For instance, the isolates (Mexico-2008-4 and Mexico-2008-5) are much closer to strains of *S. enterica* subsp. *arizonae* and even to *S. bongori* than to *S*. Infantis. Indeed, one of these Mexican isolates proved to be *S. enterica* subsp. *diarizonae* in *in silico* antigenic profile prediction. Senegal SARB27 was typed by *in silico* MLST typing as ST79 that is more likely characteristic for some exotic strains of *S*. Infantis^1^. These discrepancies between the results of traditional and whole genome sequence-based typing methods point to the need of exploiting the available *in silico* tools for strain typing in order to avoid misidentification.

Our analysis is in accordance to the data of Liu et al. (2017) that the PCR systems based on the eight selected flagellin and flagellin related gene sequences can be applicable for the detection of Infantis serovar; however, some exceptions are possible. Accordingly, in case of 76 *S*. Infantis genomes, this analysis mainly corresponds to the results of “conventional” serovar classification. However, it should be mentioned that according to our MLST analysis, the nine outlier isolates need to be reclassified.

Analyzing “non-Infantis” and “Infantis only” genomes separately confirmed that the distinguishing resolution was generally better in the Infantis only system as compared to the combined analysis on “non-Infantis” plus “Infantis.” As the *S*. Infantis genomes were missing from the analyses of Zou et al. (2013), we also wanted to get some idea about possible differentiation of *S*. Infantis from the main *Salmonella* serovars. Here we did not only confirm the results of Zou et al. (2013) about genetic distances between the tested *Salmonella* serovars and *S*. Infantis, but also confirmed our findings on genomic distances between certain lineages of *S*. Infantis.

## Conclusion

The comprehensive genome analysis based on the whole-, core-, and accessory genomes could better elucidate genetic relatedness of local and global as well as of pre-emerging and emerging strains of *S.* Infantis. Among these methods the core genome analysis has the least discriminatory power, while analysis of cloud genes offered the most detailed insight into the genetic distances and relationships of *S*. Infantis strains. Our analyses demonstrated that, contrary to the claim of Hindermann et al. (2017), the Swiss strains are not directly related to the Hungarian ones. Accordingly, in spite of the comprehensive analysis of several genomic characteristics, no direct epidemiologic links between *S.* Infantis strains from different countries could be established. Our results also suggest the need for genome-based re-classification of some *Salmonella* strains for which the sequences were originally deposited in the databases as *S*. Infantis.

## Data Availability Statement

All datasets generated for this study are included in the article/Supplementary Material.

## Author Contributions

TN, AS, and TW performed the bioinformatic analyses. JP carried out the PFGE analysis. BN and FO conceived the project. FO, BN, JK, AS, TW, TN, and MS wrote the manuscript. All authors reviewed the manuscript.

## Conflict of Interest

The authors declare that the research was conducted in the absence of any commercial or financial relationships that could be construed as a potential conflict of interest.

## Footnotes

1 http://enterobase.warwick.ac.uk/species/index/senterica
