# Supplementary material for: Comparative Genome Analysis of Hungarian and Global Strains of Salmonella Infantis
Source: Front Microbiol. 2020 Apr 3;11:539. doi: 10.3389/fmicb.2020.00539 (PMC7147451; doi:10.3389/fmicb.2020.00539)
Supplement: Supplementary file 1 [file Data_Sheet_1.PDF]

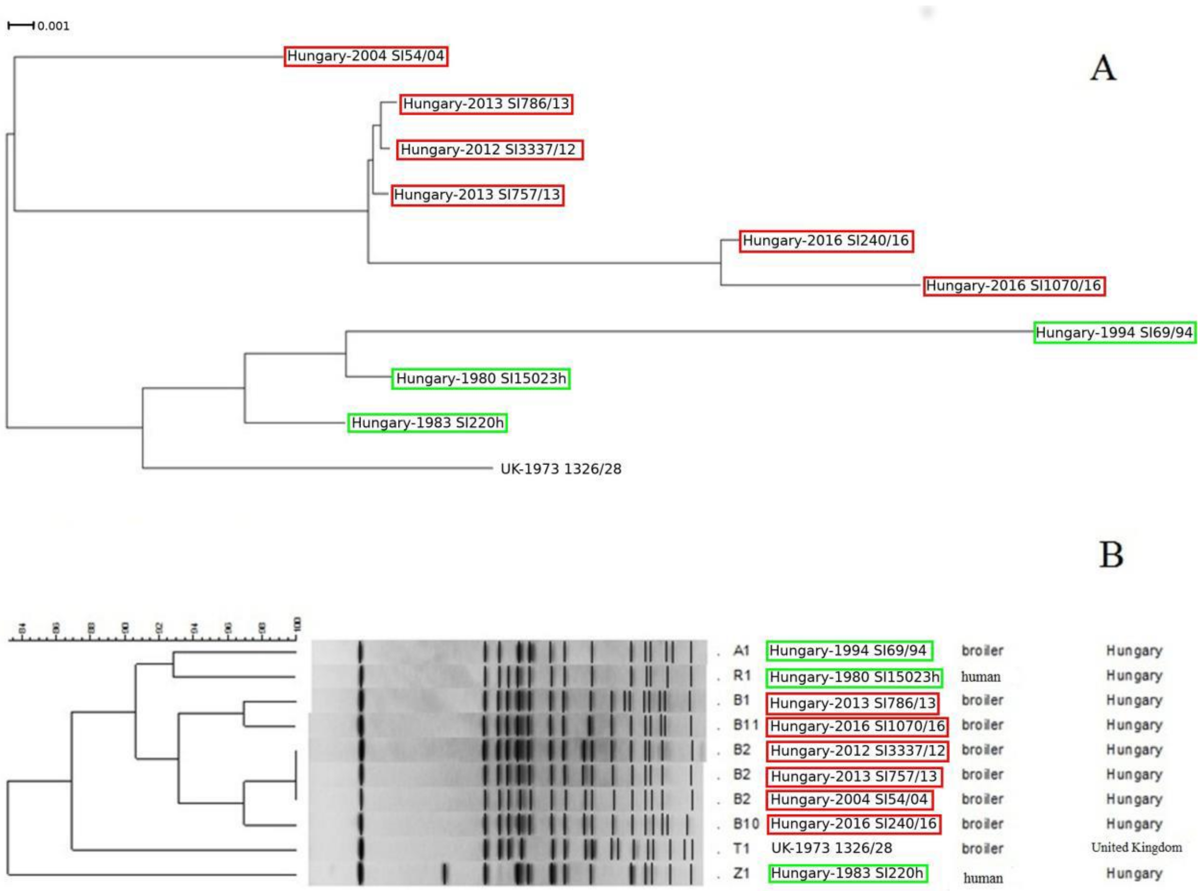

Supplementary Figure 1. (A): Whole genome tree of the 9 Hungarian- and the UK strains of *S*. Infantis. The two major clusters are for the emerging strains (red boxes), and for the pre-emerging ones (green boxes). (B): PFGE analysis of the Hungarian *S*. Infantis genomes. Emerging strains form a cluster separate from the pre-emerging isolates and from the UK strain. PFGE was carried out according to the standardized Pulsenet protocol (Ribot et al., 2006; Nógrády et al., 2012).

References:

Nógrády, Király, Davies, and Nagy (2012). Multidrugresistant clones of *Salmonella* Infantis of broiler origin in Europe. *International Journal of Food Microbiology, 157*(1), 108-112. doi:10.1016/j.ijfoodmicro.2012.04.007

Ribot, E. M., Fair, M. A., Gautom, R., Cameron, D. N., Hunter, S. B., Swaminathan, B., and Barrett, T. J. (2006). Standardization of pulsed-field gelelectrophoresis protocols for the subtyping of Escherichia coli O157: H7 Salmonella, and Shigella for PulseNet. *Foodborne Pathogens and Disease, 3*(1), 59-67. doi:10.1089/fpd.2006.3.59

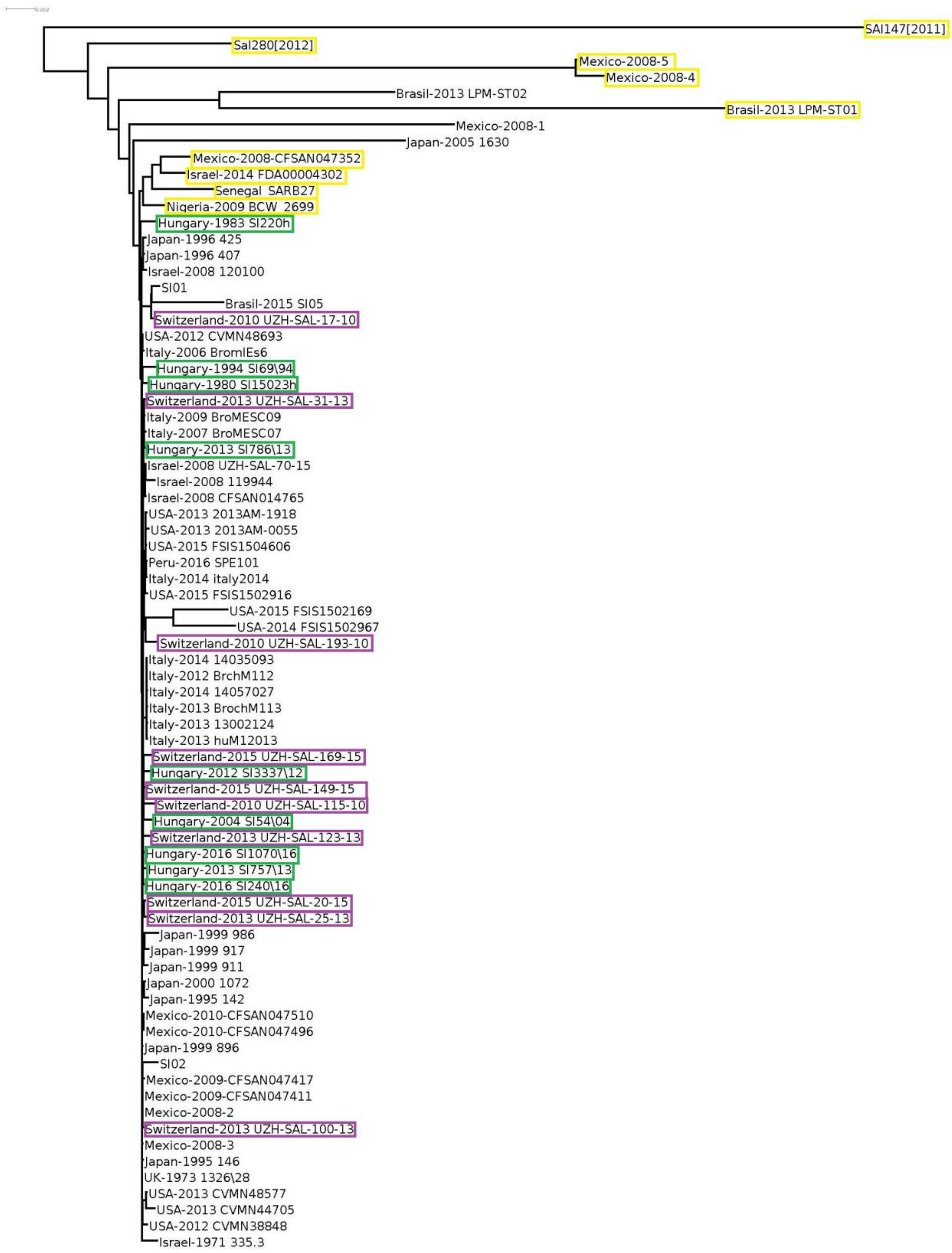

Supplementary Figure 2. Core genome tree of *Salmonella* Infantis strains investigated. Green boxes show the pre-emerging and the recent Hungarian isolates; purple boxes indicate Swiss strains, while yellow boxes indicate the *Salmonella* strains originally deposited as *S.* Infantis but outlying from the Infantis cluster. Abbreviations and symbols are as in Figure 1.
